# Development versus predation: Transcriptomic changes during the lifecycle of *Myxococcus xanthus*

**DOI:** 10.3389/fmicb.2022.1004476

**Published:** 2022-09-26

**Authors:** Juana Pérez, Francisco Javier Contreras-Moreno, José Muñoz-Dorado, Aurelio Moraleda-Muñoz

**Affiliations:** Departamento de Microbiología, Facultad de Ciencias, Universidad de Granada, Granada, Spain

**Keywords:** *Myxococcus xanthus*, *Sinorhizobacterium meliloti*, bacterial predation, development, transcriptome, predatosome

## Abstract

*Myxococcus xanthus* is a multicellular bacterium with a complex lifecycle. It is a soil-dwelling predator that preys on a wide variety of microorganisms by using a group and collaborative epibiotic strategy. In the absence of nutrients this myxobacterium enters in a unique developmental program by using sophisticated and complex regulatory systems where more than 1,400 genes are transcriptional regulated to guide the community to aggregate into macroscopic fruiting bodies filled of environmentally resistant myxospores. Herein, we analyze the predatosome of *M. xanthus*, that is, the transcriptomic changes that the predator undergoes when encounters a prey. This study has been carried out using as a prey *Sinorhizobium meliloti*, a nitrogen fixing bacteria very important for the fertility of soils. The transcriptional changes include upregulation of genes that help the cells to detect, kill, lyse, and consume the prey, but also downregulation of genes not required for the predatory process. Our results have shown that, as expected, many genes encoding hydrolytic enzymes and enzymes involved in biosynthesis of secondary metabolites increase their expression levels. Moreover, it has been found that the predator modifies its lipid composition and overproduces siderophores to take up iron. Comparison with developmental transcriptome reveals that *M. xanthus* downregulates the expression of a significant number of genes coding for regulatory elements, many of which have been demonstrated to be key elements during development. This study shows for the first time a global view of the *M. xanthus* lifecycle from a transcriptome perspective.

## Introduction

Predatory bacteria use other living bacteria or fungi as food sources, for which they actively hunt to kill them and consume their macromolecules as nutrients. These small predators are widely distributed in many natural and artificial environments where they play important roles in maintaining microbial diversity and shaping ecosystems (Chauhan et al., 2009; Chen et al., 2011; Griffin et al., 2013; Kandel et al., 2014; Johnke et al., 2017). In the last years, bacterial predators have been receiving substantial recognition and are attracting the attention of many research groups because of their potential applications as alternative therapies, in which whole cells of predators can be used as weapons to kill or control the growth of other resistant bacteria; that is, their use in bacterial therapy as “living antibiotics” or as biological resources producing innovative antimicrobials and other bioactive products (Pérez et al., 2016, 2020).

Bacterial predators can be grouped into two main hunting categories: endobiotic and epibiotic (Pérez et al., 2016). However, there are some bacterial predators that, although can act as solitary epibiotic hunters, in nature preferably attack forming multicellular groups where individual cells cooperate within the community by sharing mixtures of diffusible hydrolytic enzymes and secondary metabolites that kill and decompose the prey before consuming the released nutrients (Pérez et al., 2020; Thiery and Kaimer, 2020). This latter strategy is used by myxobacteria, which are abundant in soils and have been recently described as a key taxon in the soil food webs (Petters et al., 2021). *Myxococcus xanthus* is the most studied myxobacteria, and besides being a predator, it is also a model organism for studying prokaryotic development and bacterial multicellularity (Muñoz-Dorado et al., 2016). This bacterium moves on solid surfaces in a coordinated manner, and forms dynamic, multicellular groups called swarms, within which cells interact with each other by complex inter-and extracellular signaling systems. It moves by using two different locomotion systems, social motility (S-motility) and adventurous motility (A-motility). In natural environments, where they share niche with other microorganisms, *M. xanthus* swarms move in a coordinate way, and when they find prey, the population lyses them and absorbs their nutrients (Muñoz-Dorado et al., 2016). Under nutrient-deficient conditions, *M. xanthus* accomplishes the developmental cycle, where thousands of organized bacteria aggregate forming macroscopic structures termed fruiting bodies. During development, three subpopulations of cells show division of labor: a small fraction of cells becomes round resistant myxospores, another part remains as peripheral rods, whereas most of the cells die, probably to provide nutrients that allow cells to aggregate and differentiate. When nutritional conditions are favorable again, the myxospores in a fruiting body germinate and originate a predatory swarm (Muñoz-Dorado et al., 2016).

For many years, myxobacteriologists have concentrated their efforts on the developmental cycle, and a profound knowledge has been accumulated on the complex regulation, the intra-and extracellular signaling, and the motility mechanisms that requires this multicellular behavior (Claessen et al., 2014; Cao et al., 2015; Pérez et al., 2016). There are also many studies on the use of myxobateria as micro-factories of new products (Bader et al., 2020), and other research groups are studying complex adaptions to changing environments (Muñoz-Dorado et al., 2016; Pérez et al., 2018; Padmanabhan et al., 2021). Although the first studies on bacterial predators, more than 80 years ago, were carried out with myxobacteria, only in the last decades this trait has attracted the attention of researchers and some valuable information has been published to elucidate the systems and enzymes used by *M. xanthus* to contact and prey on a broad variety of microorganisms (for reviews see Keane and Berleman, 2016; Pérez et al., 2016; Thiery and Kaimer, 2020).

*M. xanthus* holds one of the largest genomes among prokaryotes, which encodes an uncommon high number of regulatory mechanisms and exhibits a huge biosynthetic capacity for degradative enzymes and secondary metabolites production (Goldman et al., 2006; Pérez et al., 2008; Muñoz-Dorado et al., 2019). Although there are some excellent groups studying the production of secondary metabolites and new antimicrobial agents, and several bioactive new products have been isolated and characterized (Herrmann et al., 2017; Hoffmann et al., 2018; Bader et al., 2020), many of the predicted biosynthetic gene clusters (BGCs) remain silenced or are expressed at low level under the laboratory conditions assayed. Consequently, more research is needed to exploit more efficiently the enormous potential of this predator (Pérez et al., 2020). In this sense, transcriptomic studies by using massive sequencing techniques will help to test different conditions that resemble natural environments to unlock the expression of genes coding for enzymes involved in secondary metabolites biosynthesis that could be of pharmacological or industrial interest. In fact, the expression profiles of the dynamic transcriptome in developmental conditions showed that many of these BGCs increase during development, probably to defend spores inside the fruiting bodies or to release nutrients from preys to promote germination (Muñoz-Dorado et al., 2019). Similarly, elucidation of *M. xanthus* transcriptomic changes in diverse predatory conditions and with different prey will allow not only to increase the expression of cryptic genes, but also to assign functions to hypothetical proteins.

In our group we are studying predation of *M. xanthus* on other soil bacteria that are important from a biotechnological and/or agricultural point of view, such as the antibiotic producer *Streptomyces coelicolor* and the legume symbiont, nitrogen fixing *Sinorhizobium meliloti*. We have demonstrated that the presence of *M. xanthus* induces multicellularity and over-production of the blue-color antibiotic actinorhodin on *S. coelicolor* (Pérez et al., 2011). It has also been demonstrated that during co-culture of *M. xanthus* with reference laboratory strains and field isolates of *S. meliloti* the predator exhibits two different predatory strategies and that the exopolysaccharide galactoglucan (EPS II) is the major determinant of these patterns. This study also showed that A- and S-motility are required for efficient predation in the conditions assayed (Pérez et al., 2014). Moreover, it has been revealed that copper plays an important role in *M. xanthus*-*S. meliloti* interaction, inducing in the prey the biosynthesis of melanin to protect itself against predation (Contreras-Moreno et al., 2020). Now, we are focused on the elucidation of the *M. xanthus* predatosome. The predatosome can be defined as the transcriptomic changes in the predator in response to the presence of the prey. We will use this term to refer to the upregulated genes that will help to the bacterial predator to recognize, contact, kill and lyse the prey, and use the by-products, but also to the downregulated genes that, in the case of the multicellular *M. xanthus*, are especially important, because they are in charge of diverting the complex lifecycle towards predation, avoiding development.

Herein we have used RNA-seq technology to define the predatosome of *M. xanthus* preying on *S. meliloti*. The results obtained, in addition to identify which specific genes are upregulated to detect, kill, lyse, and consume the prey, have revealed that this myxobacterium modify its lipid composition. Moreover, comparison with other transcriptomic changes during predation on other prey has allowed to outline a core predatosome. Finally, comparison with the developmental transcriptome has allowed to draw a global transcriptional perspective of the complex lifecycle of this intriguing multicellular bacterium.

## Materials and methods

### Preparation of *M. xanthus* synchronously predatory cells

*M. xanthus* strain DK1622 (Kaiser, 1979; Goldman et al., 2006) and *S. meliloti* Rm1021 (Meade and Signer, 1977) used in this study were grown in CTT (Hodgkin and Kaiser, 1979) and TY (Beringer, 1974) broth, respectively, with vigorous shaking at 30°C. The rhizobial culture was grown to an optical density at 600 nm (OD_600_) of 1 and then diluted to a final OD_600_ of 0.2. For each replicate, twenty 10-μl drops of the diluted culture were deposited on the surface of CTT agar plates (1.5% Bacto-Agar [Difco] supplemented) and incubated at 30°C for 24 h. Then, *M. xanthus* cells grown in CTT liquid media to an OD_600_ of 1 were centrifuged and concentrated in TM buffer (10 mM Tris–HCl [pH 7.6], 1 mM MgSO_4_) to a final OD_600_ of 15. Then, ten-microliters drops of concentrated culture of the *M. xanthus* strain were deposited on top of each of the rhizobial colonies (samples Mx_Sm) or twenty 10-μl drops were placed onto CTT plates (samples Mx; Supplementary Figure S1). After 2 and 6 h of incubation, two replicates from each of the two conditions (predator/prey co-culture and pure culture of *M. xanthus*) were harvested from plates, and the obtained pellets were transferred immediately into 0.5 ml of RNA Protect Bacteria Reagent (Qiagen). Cells were then incubated at room temperature for 5 min, harvested by centrifugation at 5000× *g* for 10 min (4°C), and stored at −80°C after removal of the supernatant. For the *M. xanthus* t = 0 samples (samples Mx_t0), two replicates of 3 ml of the original liquid culture (OD_600_ of 1) were harvested by centrifugation as above, resuspended in RNA Protect Bacteria Reagent, and processed in the same manner.

### RNA extraction

To isolate RNA, frozen pellets were thawed and resuspended in 250 μl of 3 mg/ml lysozyme (Roche) and 0.4 mg/ml proteinase K (Ambion) prepared in TE buffer (10 mm Tris–HCl; 1 mm ethylenediaminetetraacetic acid [EDTA], pH 8.0) for cell lysis. Samples were incubated 10 min at room temperature. RNA extraction was carried out using the RNeasy Mini Kit (Qiagen), performing on-column DNase digestion with the RNAse-free DNase set (Qiagen), and each sample was eluted in 50 μl of RNase-free water. The concentration of RNA was measured using a NanoDrop ND-2000 spectrophotometer (NanoDrop Technologies, United States). Total RNA samples were processed by Novogene (Novogene [United Kingdom] Company Ltd.) as indicated below.

### Library preparation

To obtain cDNA strand-specific libraries the NEBNext® Ultra™ Directional RNA Library Prep Kit (New England BioLabs, Inc.) was used. Briefly, after passing an initial quality control, rRNA was removed from total RNA samples with the Illumina Ribo-Zero Plus rRNA Reduction Kit (Illumina, Inc.), and remaining RNA was randomly fragmented. First strand DNA was synthetized using random hexamer primers and in the next step, second-strand DNA was synthesized. Double-stranded cDNA was purified using Agencourt AMPure XP Beads, and then end-repair, polyadenylation and adaptor-ligation processes were performed sequentially. Next, the ligation reaction was size-selected and purified using AMPure XP Beads, and the products obtained were used for PCR library enrichment. The PCR library was purified using Agencourt AMPure XP Beads. Libraries were quantified by qPCR and insert size was determined using LabChip GX Nucleic Acid Analyzer (Perkin-Elmer Inc.).

### Sequencing and global transcriptomic data analysis

The cDNA from two biological replicates of each condition (see above) was used for sequencing using the Illumina NovaSeq6000 (150 bp paired-end read) sequencing platform (Novogene [UK] Company Ltd.). Sequence reads were pre-processed to remove low-quality bases. Next, reads were mapped against *M. xanthus* DK1622 and/or *S. meliloti* Rm1021 genome sequences using Bowtie2 with the mismatch parameter set to two and other parameters set to default, using the pair-end strategy. For visualization of mapping status of reads files were provided in BAM format. Artemis v.18.0.0 (Carver et al., 2012) was used for the visualization of the sequence reads against the reference genomes. FPKM (fragments per kilobase of transcript per million fragments mapped) normalization was used for comparison of samples. The average FPKM values of the two replicates were used to calculate the log2 fold change.

On average, 16.16 million read pairs and a coverage of 266X were obtained. After removing the ribosomal sequences, the genome coverage varied from 5.52 to 14.18x (median of 10.49x), enough to provide an adequate coverage of the mRNA fraction.

The DESeq2 R/EdgeR R package was used to identify differentially expressed transcripts (Robinson et al., 2010; Love et al., 2014).

## Results and discussion

### Global data of the early transcriptomic response of *M. xanthus* on predatory co-cultures

When the predator contacts with the prey it must quickly and precisely adapt to the new nutrient source that entails a living microorganism. The response must be specific and will reveal the mechanism used for predation. Here, the early response of *M. xanthus* to the prey *S. meliloti* has been analyzed by collecting co-cultures samples at 2 and 6 h. We have previously reported that at later times most prey cells are lysed (Pérez et al., 2014). At 2 h the predator has already established the first contact with the prey and is actively attacking. Comparison with a longer time (6 h) will allow us to know which genes are important in the first attack and which are continuously needed during predation.

To examine transcriptional changes, RNA-seq technology was used. In this study, five cDNA libraries were constructed: Mx_t0: *M. xanthus* alone at time 0 h; Mx_t2: *M. xanthus* alone collected after 2 h in solid CTT medium; Mx_t6: *M. xanthus* alone grown for 6 h in solid CTT medium; Mx_Smt2: cells collected after 2 h of interaction *M. xanthus*-*S. meliloti*; and Mx_Smt6: where cells were harvested after 6 h of interaction *M. xanthus*-*S. meliloti*. For simplicity, we will term t2 and t6 the results obtained at 2 and 6 h of the co-cultures of *M. xanthus* and *S. meliloti* (Mx_Smt2 and Mx_Smt6, respectively) compared to their respective controls (predator cells in pure culture at 2 or 6 h: Mx_t2 and Mx_t6, respectively).

The total raw reads generated from each sample, the clean reads obtained after removal from the raw reads those containing adapters and/or low quality, the errors Q20 and Q30, and GC content of the clean reads are summarized in Supplementary Table S1A.

Comparison between the two replicates of each condition showed a Pearson correlation coefficient (*R*^2^) close to 1 (all ≥0.947; Supplementary Figure S2A). The median of both values was used for further analyses. To establish relationships between gene expression profiles of all the samples, we performed a principal component analysis (PCA) analysis. As expected, genes of the same condition cluster together and genes of different nutritional stage and times cluster separately (Supplementary Figure S2B). The mRNA mean reads were normalized to FPKM values (Supplementary Table S1B). Comparisons of FPKM values between samples exhibited similar mRNA expression levels and the violin diagram of FPKM visually showed the gene expression levels (Supplementary Figures S2C,D). All these data show that the transcriptome is of good quality.

In our comparison analysis we will use the old nomenclature (MXAN_), since it is most frequently used in the literature. However, in all the tables the corresponding new localizers MXAN_RS are also indicated.

### Functional enrichment and differentially expressed genes (DEGs) in co-culture

**Figure 1:**
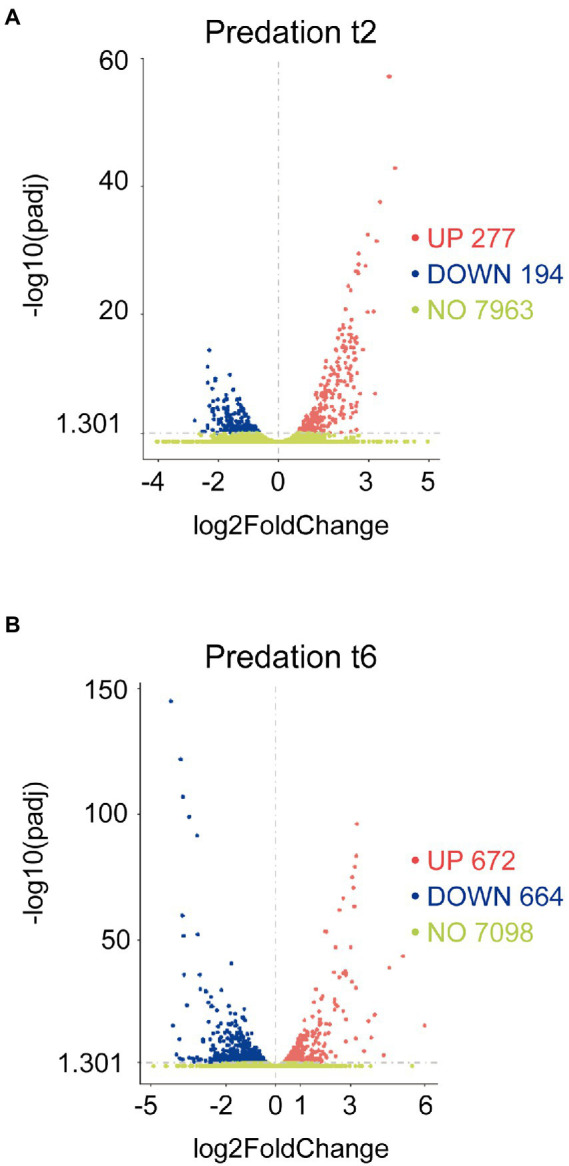
Differential expression response of *M. xanthus* during predation. Screening of differentially upregulated **(A)** and downregulated **(B)** genes by volcano at t2 and t6 in predatory conditions. Volcano plots show the estimated fold changes (*x*-axis) versus the minus log10 of the adjusted *p*-values (*y*-axis) from DEseq analysis. Significant genes with absolute values of |Log2 Fold Change|>0 and *p*adj<0.05 are shown in red (upregulated) or in blue (downregulated). Black vertical dotted line indicates zero-fold change. Green dots indicate non-regulated genes.

The transcripts of co-cultures at 2 and 6 h versus their respective controls were filtered by their fold changes (|Log2 Fold Change| > 0) and *p*adj<0.05 to construct the volcano plots to infer the overall distribution (Figures 1A,B). In total, we detected that 277 at 2 h and 672 at 6 h transcripts were upregulated (including sRNAs, i.e., non-coding RNA of 50–500 nt length), while 194 at 2 h and 664 at 6 h were downregulated (Supplementary Tables S1C,D).

To elucidate the biological processes associated to DEGs during predation, enrichment analyses were carried out using the associated pathways in the KEGG database (Kyoto Encyclopedia of Genes and Genomes; Kanehisa et al., 2021). The analysis of upregulated genes showed that at 2 h (Mx_Smt2 vs. Mx_t2) the profiles of significantly enriched functions and pathways change drastically compared to 6 h (Mx_Smt6 vs. Mx_t6; Supplementary Figure S3A; Supplementary Tables S1E,F). At 2 h, the predominant pathways are involved in fatty acid (FA) metabolism, secondary metabolite biosynthesis, oxidative phosphorylation, and two-component systems (Supplementary Figure S3A). However, at 6 h, cells prioritize translation, since many induced genes code for structural constituent of ribosomes, peptide biosynthesis, tRNA, and protein metabolism (Supplementary Figure S3A).

Significantly pathways detected among downregulated genes at 2 and 6 h are those involved in two-component systems and bacterial chemotaxis (Supplementary Figure S3B; Supplementary Tables S1G, H).

All these DEGs at 2 and 6 h were further manually analyzed and compared to the literature (Supplementary Tables S2A,B). We have excluded novel genes and sRNAs in these studies to compare our data with the actual knowledge about the lifecycle of *M. xanthus*. We have also considered in our analyses genes that are predictably encoded in the same cluster or in the same operon according to the locations of reads in the reference genome, transcription start sites, and transcription termination sites of operons by using Rockhopper system (Tjaden, 2020).

For a better picture of the global changes that take place during co-culture and to decipher the predatosome, we have considered all the up and downregulated genes with |Log2 Fold Change|>0 and *p*adj<0.05. The reason is that we are comparing predation versus growth on rich media and, therefore, any change in gene expression, no matter how small, must be considered, because the differences must be attributed to the presence of living prey as nutrients. It has been also taken under consideration in our analysis those genes that are in operons or clusters, or those which functions or implications in different metabolic pathways have been previously described. For improved confidence, a threshold of Log2 Fold Change|>1 is indicated in the all the figures. However, it should be considered that some defensive or antagonistic responses of *S. meliloti* against *M. xanthus* would impact gene expression of the predator during the interaction. This interactive response is under research in our laboratory.

#### Hydrolytic enzymes and extracellular proteins

**Figure 2:**
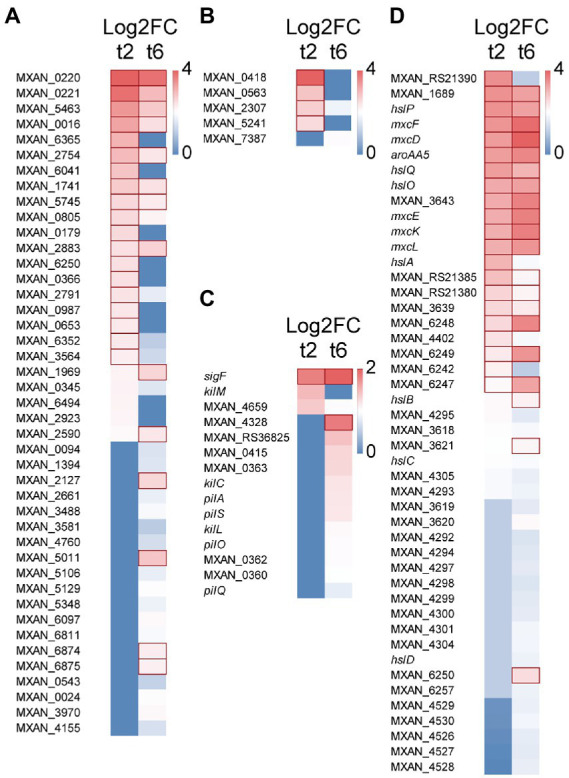
Heatmap with upregulated genes during predation at t2 and/or t6. **(A)** Hydrolytic enzymes. **(B)** LysM proteins. **(C)** S-motility. **(D)** Secondary metabolites. Red edges indicate genes with Log2 Fold Change > 1.

Forty-four genes coding for hydrolytic enzymes were upregulated with Log2 Fold Change from 0.78 to 3.4 (Figure 2A): 20 peptidases, 5 alpha-beta fold hydrolases, 5 nucleases, 3 lipases, 3 lactamases, 3 amidases, 1 phosphoesterase, 1 sulfatase, 1 thioesterase, 1 polysaccharide lyase, and 1 poly-hydroxyalkanoate depolimerase. Most of these enzymes have never been studied, although some of them have been associated to predation. Thus, the peptidase MepA has been suggested to be used as a secondary factor during predation, to break down proteins released by already lysed cells (Berleman et al., 2014); 3 other peptidases have been detected in an extracellular fraction of *M. xanthus* that showed bacteriolytic activity (Arend et al., 2021); and 5 other hydrolytic enzymes have been experimentally located in outer membrane vesicles (OMV): MXAN_0220, MXAN_2661 (Kahnt et al., 2010), MXAN_0976 (Zwarycz et al., 2020), MXAN_0366, and MepA (Berleman et al., 2014).

On the other hand, other 7 genes code for proteins with no hydrolytic activity, but located or enriched in OMVs, such as the outer membrane protein Oar (Martínez-Cañamero et al., 1993), which is a TonB-dependent transporter involved in secretion of proteins (Gómez-Santos et al., 2019). Since Oar is the major protein of the outer membrane of *M. xanthus* (Martínez-Cañamero et al., 1993), it is plausible to speculate that it may play an essential role in secretion of factors required for predation of bacteria, similarly as it has been reported for the predation of fungi by myxobacteria (Li et al., 2019).

Moreover, 5 genes encode proteins holding peptidoglycan-binding LysM domains (Figure 2B). These domains have been found in a wide variety of extracellular proteins and receptors which are responsible for detection of and binding to peptidoglycans, and even they are able to sense and discern between different microorganisms (Wong et al., 2019). For instance, LysM forms part of the legume sensory system NFP (Nod Factor Perception) involved in specifically detect its symbiont bacteria *S. meliloti* (Bensmihen et al., 2011). Similarly, it is expected that these *M. xanthus* proteins may be involved in recognizing specifically the peptidoglycan of the prey and it is not ruled out that they are able to discern between different microorganisms.

These results show the extraordinary capacity of *M. xanthus* to recognize, bind and lyse the prey, and open the possibility to discover new products.

#### S-motility and Tad-like apparatus

Several genes coding for proteins related to *M. xanthus* S-motility are slightly upregulated at 6 h (Log2 Fold Change from 0.33 to 1.31), including the *sigF* sigma factor, which is involved in the regulation of genes for S-motility (Ueki et al., 2005; Youderian and Hartzell, 2006). Several genes found in this study are in the same operon (MXAN_0360–0363; Fremgen et al., 2013) or in the same cluster (*pilA*, *pilO*, *pilS*, *pilQ*; Wall and Kaiser, 1999). On the other hand, 4 genes coding for the Tad-like apparatus (Kil proteins) are upregulated either at 2 or 6 h (Log2 Fold Change from 0.4 to 0.93): MXAN_3106 (*kilC*) and MXAN_4658–4660 (*kilL, kilH* and *kilM*), which have been recently described to be involved in contact-dependent prey killing (Seef et al., 2021; Figure 2C). These results are in good agreement with experimental data that have demonstrated that S-motility (Pérez et al., 2014) and the Tad-like system (Seef et al., 2021) are both important for predation.

#### Secondary metabolites

*M. xanthus* produces a great variety of biologically actives metabolites. However, only a few of them have been implicated in predation. Moreover, many genes potentially coding for enzymes involved in secondary metabolites biosynthesis are cryptic in laboratory conditions. In this analysis, it has been found that during the intertaction with the prey one cluster of genes consisting of 3 non-ribosomal peptide synthetases and 1 hybrid non-ribosomal peptide synthetase/type I polyketide synthase (MXAN_RS21375-MXAN_RS21390*)* is upregulated (Log2 Fold Change from 1.1 to 2.6) and, consequently, it must be involved in the biosynthesis of an unidentified bio-product. A second cluster (MXAN_3618–3620) is implicated in the biosynthesis of the siderophore myxochelin (Korp et al., 2016, 2018; Figure 2D; Supplementary Table S2A). In addition, 5 type I polyketide synthases located in the myxalamide cluster are also upregulated at 6 h (MXAN_4526-MXAN_4530), suggesting that this antibiotic is also required for killing the prey, although further research is required. Similarly, DK-xanthene also seems to play a role in predation because 10 genes of the operon are also slightly upregulated (Figure 2D; Supplementary Table S2A). These results agree with the finding that DK-xanthene is overproduced during predation (Ellis et al., 2019). Finally, it is interesting to mention that genes encoding enzymes involved in the biosynthesis of the antibiotics myxovirescin and myxoprincomide are not upregulated in the interaction *M. xanthus-S. meliloti*, although these two antibiotics have been reported to be involved in predation of *M. xanthus* on *E. coli* (Xiao et al., 2011) and *B. subtilis* (Müller et al., 2016). Therefore, it remains to be elucidated whether these antibiotics are overproduced only on specific prey, or whether their overproduction is regulated by a post-transcriptional mechanism. An interesting result is the upregulation (Log2 Fold Change from 0.6 to 2.7) of genes involved in geosmin biosynthesis (MXAN_6242–6257), including genes coding for a terpene synthase and two transcriptional regulators of the Crp/Fnr family, suggesting that this secondary metabolite somehow participates in predation (Figure 2D).

Another remarkable result is the upregulation (Log2 Fold Change from 0.6 to 2.6) of a cluster (*hslA*, *hslB*, *hslC*, *hslD*, *hslO*, *hslP*, *hslQ*), involved in the synthesis of homospermidine lipids (Hoffmann et al., 2018; Figure 2D). Homospermidine lipids are modified polyamines formed during *M. xanthus* development, which are bioactive against a panel of microorganisms (Hoffmann et al., 2018). Our data indicate that they also participate in predation. Hoffmann et al. (2018) proposed that these secondary metabolites are originated from arginine, *via* the putrescine pathway. The fact that the complete cluster (MXAN_5105–5110) involved in arginine biosynthesis is also upregulated in predatory conditions reinforces this suggestion (Supplementary Table S2A).

These data open the door to identify new secondary metabolites that may have biotechnological applications. Moreover, they will help to find new roles for secondary metabolites that have already been identified.

#### Lipid metabolism

**Figure 3:**
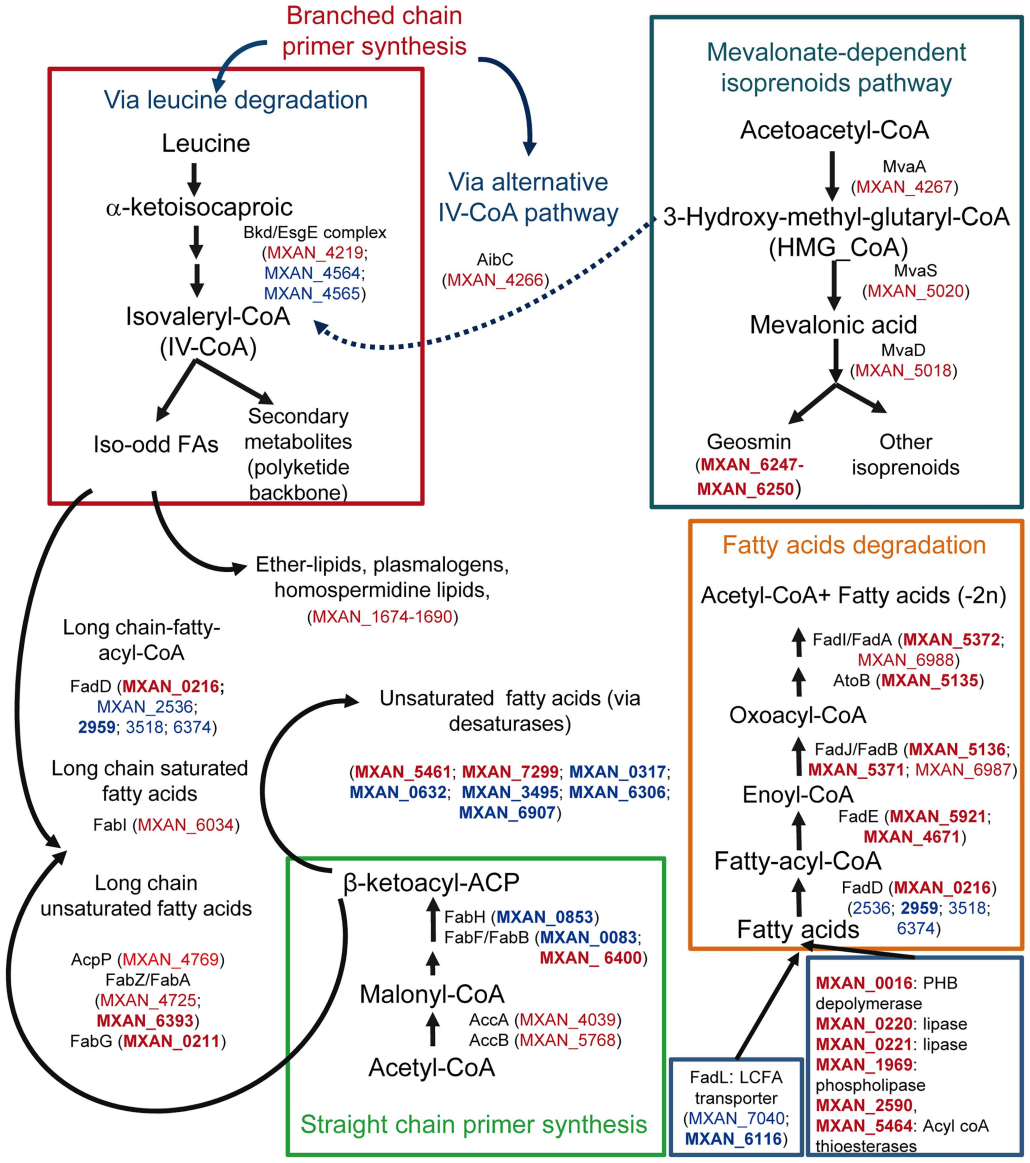
Differential expression during predation at t2 and t6 of genes involved in lipid biosynthesis and degradation, and their possible implication in lipid external use, and secondary metabolites biosynthesis. Red text indicates genes upregulated during predation. Blue text indicates downregulated genes. In bold, |Log2 Fold Change|> 1.

Many genes involved in FA metabolism are also upregulated during co-culture. It is notable to mention straight-chain FA biosynthesis, since many genes involved in their synthesis are upregulated (Figure 3; Supplementary Table S2A). However, MXAN_0853, whose inactivation blocks completely the production in *M. xanthus* of straight-chain FA (Bode et al., 2006a), is downregulated at 2 h (Supplementary Table S2B). It could be argued that gene MXAN_0215, which exhibits high similarity to MXAN_0853 and is upregulated, would carry out the same function (Figure 3), but it has been reported that its disruption has no effect in lipid composition (Curtis, 2001; Bode et al., 2006a). Consequently, MXAN_0215 might have complementary functions with respect to starting unit specificity and might be involved in the formation of iso-FA during predation.

Some other upregulated genes are implicated in the formation of isovaleryl-CoA (IV-CoA), which is the primer for branched-chain iso-odd FA biosynthesis. These FA are responsible for maintaining membrane fluidity, and are involved in signaling during development and in biosynthesis of some secondary metabolites (Bode et al., 2006b; Li et al., 2013). *M. xanthus* can synthesize IV-CoA *via* leucine degradation, but also from 3-hydroxy-methyl-glutaryl-CoA (HMG_CoA) *via* an alternative system (*aib* operon) from the mevalonate pathway (Figure 3; Bode et al., 2009; Li et al., 2013; Okoth et al., 2020). As observed in Figure 3, some genes of the Bkd/Esg complex (branched-chain α-keto acid dehydrogenase complex/E signal) involved in IV-CoA *via* leucine degradation are downregulated, but genes from the mevalonate pathway, including the *aibC* gene, which converts HMG-CoA into IV-CoA, are upregulated. This alternative pathway is essential for fruiting body formation, presumably to supply IV-CoA when leucine is limited (Bode et al., 2006b). Until now, there are no evidences that this alternative pathway functions during growth. However, our results indicate that this route is operative also in predatory conditions (Figure 3). On the other hand, Pasternak et al. (2013), comparing the genomes of predatory and non-predatory bacteria, found that they differ in isoprenoid biosynthesis. While all predators used the mevalonate pathway, the non-mevalonate pathway is used by non-predatory bacteria. Our results reinforce the idea that this mevalonate route might be important for bacterial predation. Furthermore, the mevalonate pathway is also involved in geosmin biosynthesis in *M. xanthus* (Bode et al., 2009; Korp et al., 2016), indicating again a role of this volatile isoprenoid in predation.

Many genes coding for FA-elongation enzymes, related to saturated FA biosynthesis (Curtis and Shimkets, 2008), are also upregulated in the presence of the prey, reinforcing the idea that lipid synthesis is activated during predation, most likely to build blocks for predator membranes. During growth *M. xanthus* incorporates predominantly unsaturated FA at *sn*-1 of the phospholipids (Curtis et al., 2006). The source of these unsaturated FA in *M. xanthus* is unknown, although desaturases must be involved. However, during predation, many genes that code for desaturases are significantly downregulated (Figure 3; Supplementary Table S2B), suggesting that *M. xanthus* is changing the composition of the membranes in the cell envelope in the presence of the prey, incorporating more saturated FAs. It is tempting to speculate that these changes may be necessary in the predator to defend from its own arsenal used during predation.

Although ester bonds are the most common linkages in phosphatidylethanolamine in *Bacteria*, phospholipids containing ether-linked chains (ether lipids) are prevalent in myxobacteria (Curtis and Shimkets, 2008). Moreover, only the branched-chain FA iso-derivatives are found in ether-linked lipids (Ring et al., 2009). These ether lipids, including plasmalogens, are responsible for membrane fluidity and confer stability to the cell against environmental stresses (Curtis and Shimkets, 2008; Gallego-García et al., 2019). Some genes coding for enzymes required for ether lipid synthesis in *M. xanthus*, such as MXAN_1675 and MXAN_1676, are upregulated during predation. However, since these genes also seem to be involved also in the biosynthesis of homospermidine lipids, which have antibacterial properties (Hoffmann et al., 2018), their role in predation remains to be uncovered.

Genes encoding enzymes involved in β-oxidation pathways are also upregulated, except for the enzymes that catalyze the first step and the fatty acyl-CoA ligases (FACL). FACLs activate FA before they can be assimilated into various metabolic pathways by converting FA to FA-acyl-CoA. These bioactive FA metabolites, in addition to serving as substrates for β-oxidation and phospholipid biosynthesis, are involved in protein transport, enzyme activation, protein acylation, cell signaling, and transcriptional control (Weimar et al., 2002; DiRusso and Black, 2004). In *M. xanthus* there are at least 6 FACL paralogues. Only one of them, MXAN_0216, is upregulated, while four of them are downregulated and the one remaining does not change its expression levels (Supplementary Figure S4A). FACLs appear to be metabolic signals for FA degradation by bacteria in general (Weimar et al., 2002), and the fact that MXAN_0216 expression is only detected during predation suggests that this FACL might have a functional role in the transmembrane movement and activation of exogenous specific FA before consuming them as carbon or energy sources. According to the idea that *M. xanthus* uses prey lipids as nutrients is the upregulation of genes involved in lipid degradation, such as those coding for lipases or esterases (Figure 3). However, the classical long-chain FA transporters (FadL-like) are downregulated (MXAN_7040 and MXAN_6116) and, consequently, there must function in another unknown uptake process.

These data indicate that certain pathways involved in lipid metabolism are upregulated during co-culture. It is expected that anabolic pathways will provide the predator with energy. In contrast, anabolic pathways seem to be used to change the lipid composition of the cell envelope and to synthesize new secondary metabolites that will contribute to kill the prey.

#### Iron uptake

**Figure 4:**
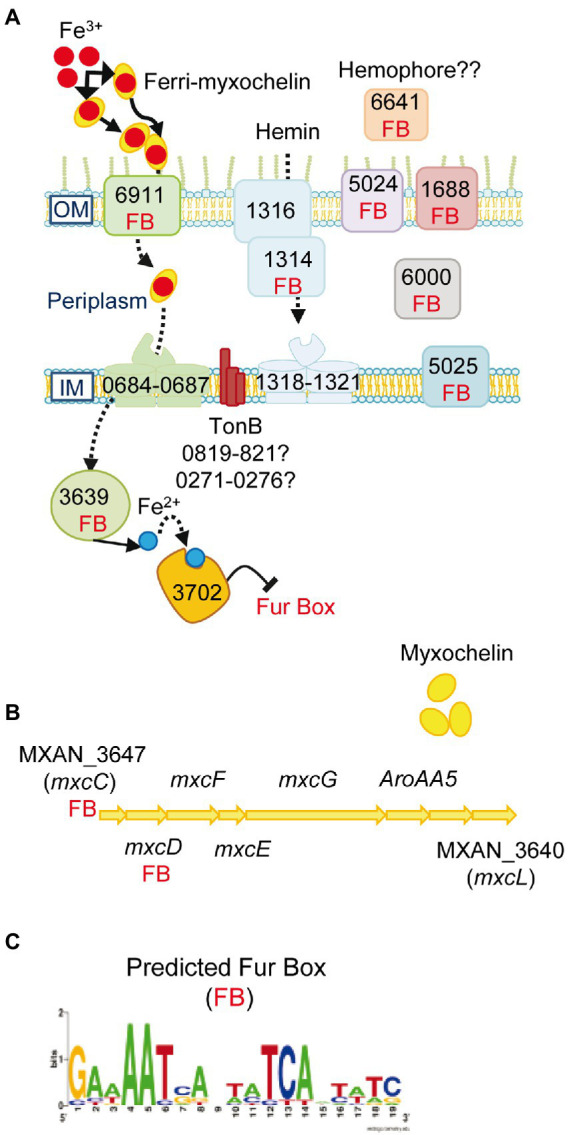
Iron uptake mechanisms are induced in co-culture. **(A)** Genes predicted to be involved in iron uptake and that are upregulated in co-culture at t2 and/or t6. The numbers of the genes depicted in the figure are the corresponding MXAN_ identifiers. **(B)** Graphical representation of the cluster of genes involved in myxochelin biosynthesis. The seven upregulated transcripts are indicated by their corresponding gene name. **(C)** Fur box (FB) predicted by using the Virtual Footprint database. Red balls: Fe^3+^; blue balls: Fe^2+^. OM, outer membrane; IM, inner membrane. For more details please see the text and Supplementary Table S2A.

Genes involved in synthesis and secretion of the high-affinity iron-chelating siderophores, myxochelin A and myxochelin B (Silakowski et al., 2000; Li et al., 2008), a TonB-dependent transporter (FepA-like; MXAN_6911) that recognizes and imports ferric siderophores, and a Fe-ABC periplasmic iron compound binding protein transporter (MXAN_0684–0687) are all upregulated by *M. xanthus* when prey on *S. meliloti*. Moreover, the siderophore reductase (MXAN_3639) that reduces Fe^3+^ to Fe^2+^ in the cytoplasm is also upregulated (Figure 4; Supplementary Table S2A). These data indicate that *M. xanthus* needs a high iron concentration and, consequently, upregulates the iron uptake machinery probably to compete for the metal in the presence of prey. The need for iron of *M. xanthus* during co-culture is reinforced by the upregulation of genes coding for heme-carrier proteins implicated in acquisition of iron from other organisms’ iron-binding proteins (MXAN_1314–1321). The TonB complexes, that energize the outer membrane receptors, are most likely MXAN_0819–0821 or MXAN_0273–0276 since some of these genes are uniquely expressed in predatory conditions. Other iron-related genes coding for proteins that have been previously detected in a proteome in iron-poor conditions or that bind to the predicted global regulatory repressor Fur (MXAN_3702) by pull-down technique (Altmeyer, 2010), are also upregulated (Supplementary Table S2A). In addition, our search for Fur boxes in *M. xanthus* genome using the Virtual Footprint database (Münch et al., 2005) against the Fur matrix of *Escherichia coli*, *Helicobacter pylori*, *Klebsiella pneumoniae*, *Vibrio cholera* and *Pseudomonas aeruginosa*, plus a manual search, has detected eighteen upregulated genes with putative Fur promoters (Figure 4; Supplementary Table S2A). Maybe this iron necessity is to supply this metal as a cofactor of enzymes required for proper predation. In fact, at least 14 genes that code for iron–sulfur proteins are upregulated during the predatory process. Alternatively, iron might be used by *M. xanthus* as a weapon for killing during predation, as it has been reported with copper (Contreras-Moreno et al., 2020). Although iron has been related with phase variation in *M. xanthus* (Dziewanowska et al., 2014), we have not observed any changes during the interaction of the myxobacterium on *S. meliloti*.

#### Ribosomal biogenesis and translation

According to the enrichment analysis, at 6 h many genes coding for proteins involved in translation are upregulated during co-culture, including genes that code for ribosomal proteins (17 genes for 30S proteins and 27 genes for 50S proteins; Supplementary Figure S4B), elongation factors (8), tRNA ligases (15), and termination factors (2) (Supplementary Table S2A). This result indicates that cells at 6 h are starting to actively synthetize proteins. At this time, predator cells have more nutrients available, and it is expected that they increase their growth rate. In addition, they must continue synthesizing proteins and secondary metabolites to lyse the prey.

#### Transcriptional regulators

It has been observed that only 24 genes coding for transcriptional factors are upregulated during co-culture (Supplementary Figure S5A; Supplementary Table S2A). These regulatory elements are expected to modulate the expression of genes that facilitate killing and lysing of the prey. In contrast, the number of genes coding for regulatory proteins that are downregulated rises to 97 (Supplementary Figure S5A; Supplementary Table S2B). Interestingly, many of these regulators have been previously implicated in the developmental cycle (Supplementary Figure S5A; Supplementary Table S2B). The relevance of these changes in regulation will be discussed below.

#### Other upregulated genes

In addition to the genes and functions reported above, it is noteworthy to mention other genes that seem to be relevant during the predatory process. For instance, 15 genes encode proteins that form part of ABC transporters (eight of them involved in iron uptake). Furthermore, 3 genes belong to the Major Facilitators Superfamily (MFS), probably involved in the export of hydrolytic enzymes or secondary metabolites. Finally, two clusters related to cytochrome c oxidase cbb3-type biogenesis, members of the heme-Cu oxidase superfamily (MXAN_5538–5541 and MXAN_5553–5557), are upregulated with the highest Log2 Fold Change obtained in this transcriptome (Supplementary Table S2A). Since those terminal oxidases are specifically required at low oxygen tensions (Pitcher and Watmough, 2004), it is tempting to speculate that the contact with the prey creates a microaerophilic atmosphere that would require of proteins that work more efficiently in these conditions.

### Searching for the core *M. xanthus* predatosome

The interaction *M. xanthus*-prey seems to differ from one prey to another, although the prey range cannot be directly correlated with the prey phylogeny (Livingstone et al., 2017; Arend et al., 2021). For instance, the strategies used by *M. xanthus* for predation on several strains of *S. meliloti* are different depending on the galactoglucan production (Pérez et al., 2014). However, there are also some common features, like the recently described Tad-like machinery that seems to be essential for predation on different preys (Seef et al., 2021).

*M. xanthus* predatosomes available so far have been obtained not only using different prey, but also under different laboratory conditions, so it is difficult to draw conclusions about common transcriptional changes that may occur when the predator confronts the prey. Some transcriptomic changes might be due to diversity of conditions, such as nutrients in the culture media used, and not only to the presence of different prey. Moreover, in our study, all predator cells are preying synchronously, while in the other reported transcriptomes predator cells are in different growth stages. Despite these difficulties, it is worth to explore the transcriptional adaptations of *M. xanthus* on different microorganisms, aiming to find common genes that can conform the core predatosome.

We first compared the transcriptome obtained on *S. meliloti* predation with those obtained on *E. coli* (Livingstone et al., 2018) and *S. coelicolor* (Lee et al., 2020). The transcriptomes obtained with live and dead *E. coli* cells were carried out in liquid medium instead of on solid medium. When live *E. coli* cells were used as prey in starvation conditions by using media with only buffer, only three genes, corresponding to the *kpdAB* system, which is involved in adaptation to osmotic shock, were upregulated, suggesting an indirect sensing of the prey. This system is not upregulated in the transcriptome on *S. meliloti*. Comparison with the predatosome against dead *E. coli* revealed 36 common genes, including 1 coding for a protein with a PilZ domain (MXAN_4328), 2 proteins with LysM domain, 2 hydrolytic enzymes, and 1 Crp/Fnr family transcriptional regulator in the geosmin cluster mentioned above (Supplementary Table S3A).

The predatosome with *S. coelicolor* (Lee et al., 2020) was performed on solid medium, where cultures of *S. coelicolor* and *M. xanthus* were spotted next to each other. Therefore, the predator must first glide for several hours to reach the prey colony. For this reason, the authors harvested the first sample at 72 h. Consequently, in their samples there are a mixture of cells actively advancing to the prey but not yet in contact, cells preying, and cells consuming the subproducts. Moreover, it should be mentioned that most nutrients included in the rich medium would be consumed by the time the predator collide with the prey. As mentioned in Material and Methods, in the transcriptome obtained in this report, cells of the predator were directly spotted on the prey. Consequently, predator enters in contact with the prey immediately and cells at different time points are at the same stage of predation. For comparison with the *S. coelicolor* predatosome, we have used only time 72 h, which we view as the most similar to our time points.

**Figure 5:**
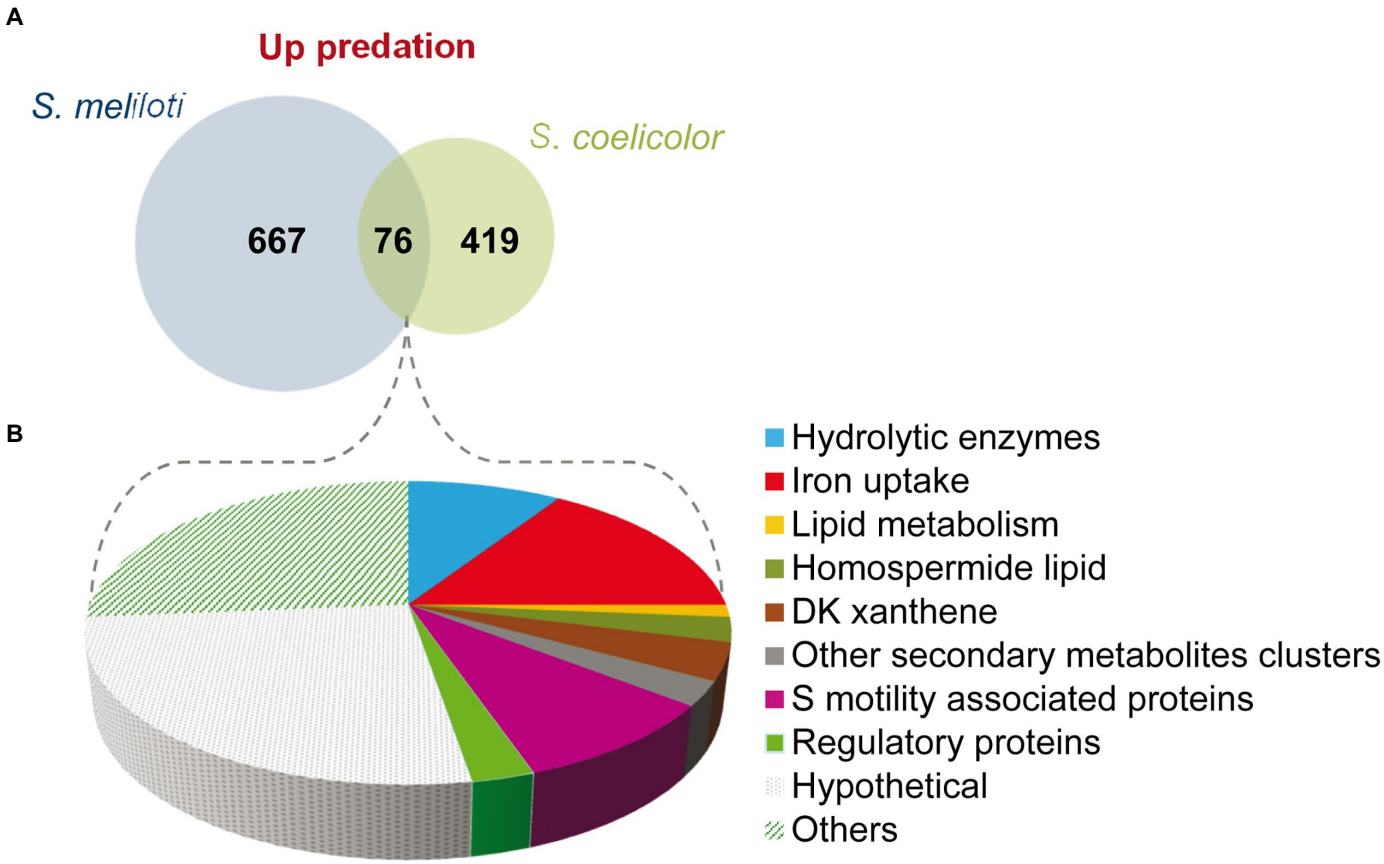
Comparison of the predatosomes of *M. xanthus* on *S. meliloti* and *S. coelicolor.* **(A)** Venn diagram of upregulated genes with Log2 Fold Change>0 and *p*adj<0.05. **(B)** Proportion of different pathways and protein functions of the 76 common genes.

A total of 76 genes have been found to be upregulated in both predatosomes, which include genes for hydrolytic enzymes and other involved in lipid metabolism, iron uptake, and motility (Figure 5; Supplementary Table S3B). However, the expression profiles of genes encoding proteins involved in the synthesis of secondary metabolites are different, such as the above mentioned upregulated clusters in *M. xanthus*-*S. meliloti* interaction involved in NRPS/PKS (type I), which are not upregulated in the interaction with *S. coelicolor* (see Section “Secondary metabolites”).

Regarding downregulated genes in both transcriptomes, only 11 were common, including a gene coding for the histidine kinase MXAN_6994, which is involved in development (Shi et al., 2008) and *katE* gene, which codes for a catalase (Thomas et al., 2008; Supplementary Table S3B).

These observations suggest that the predatosomes against *S. coelicolor* and *S. meliloti* conserve some common features, such as the modification in lipid metabolism, iron uptake, and motility, but the profiles for genes encoding enzymes involved in secondary metabolites or hydrolytic enzymes differ. Moreover, downregulated genes are also dissimilar. It should be reminded that these differences may be due not only to characteristics of the prey, but also to differences in the state of predation at which predatory cells were harvested and to the culture conditions.

To expand our understanding of the core predatosome of *M. xanthus* we have compared our data with other genes that have been reported in the literature to participate in the predatory activity of *M. xanthus*. For instance, Müller et al. (2016) carried out experiments with a collection of transposon insertion mutants and reported a number of genes required to either enhance or diminish the capacity of *M. xanthus* to consume *Bacillus subtilis.* Only four of the upregulated genes in the interaction *M. xanthus*-*S. meliloti* are also implicated in *B. subtilis* predation. Two of them code for the two-component system *hsfA*-*hsfB*, which is involved in motility, fruiting body formation, and production of secondary metabolites (Volz et al., 2012). Another common gene is in the cluster of the Tad-like apparatus described by Seef et al. (2021), and the fourth gene encodes a hypothetical protein (Supplementary Table S3C). Moreover, some hydrolytic enzymes that are upregulated in our predatosome have been previously found in OMV, in hydrolytic extracts, or have been directly implicated in predation, such as MepA (see Section “Hydrolytic enzymes and extracellular proteins”) and GroEL2, which is implicated in cell predation and macromolecular feeding (Li et al., 2010; Wang et al., 2014; Supplementary Table S3C). Although our analysis shed some light on the elucidation of the core predatosome of *M. xanthus*, new transcriptomic studies will be required using different preys and the same predatory condition to undoubtedly determine which genes are common against all preys, or at least against a wide variety of prey, and which genes are specific. These studies will also help to understand the interactions between bacterial communities in the soil.

### Completing the *M. xanthus* lifecycle from a transcriptomic point of view

As mentioned in the Introduction, *M. xanthus* exhibits a complex lifecycle comprising two stages, growth (facultative predation) and development, which are depending on the nutrient availability. To obtain a global perspective of the transcriptome dynamic along the entire lifecycle of *M. xanthus* we compare the transcriptome obtained during predation (nutrients are available and cells grow) in this study with those obtained during development (starvation conditions). So far, three *M. xanthus* developmental transcriptomes have been reported (Muñoz-Dorado et al., 2019; McLoon et al., 2021; Sharma et al., 2021), plus one about a comparison between peripheral rods versus stationary and vegetative cells (Whitfield et al., 2020). Although there are many similarities between all the developmental transcriptomes, there are also many differences. Taking into consideration that culture conditions used in the different studies were not the same, we decided to use the developmental transcriptome obtained by Muñoz-Dorado et al. (2019) to compare with the transcriptome obtained about predation on *S. meliloti*. One of the reasons is that both transcriptomes were obtained with cells cultured on solid medium, and not in submerged cultures or a flow-cell bioreactor. Moreover, cells were grown in the same conditions before they were spotted for development or predation, and concentrated at the same optical density.

**Figure 6:**
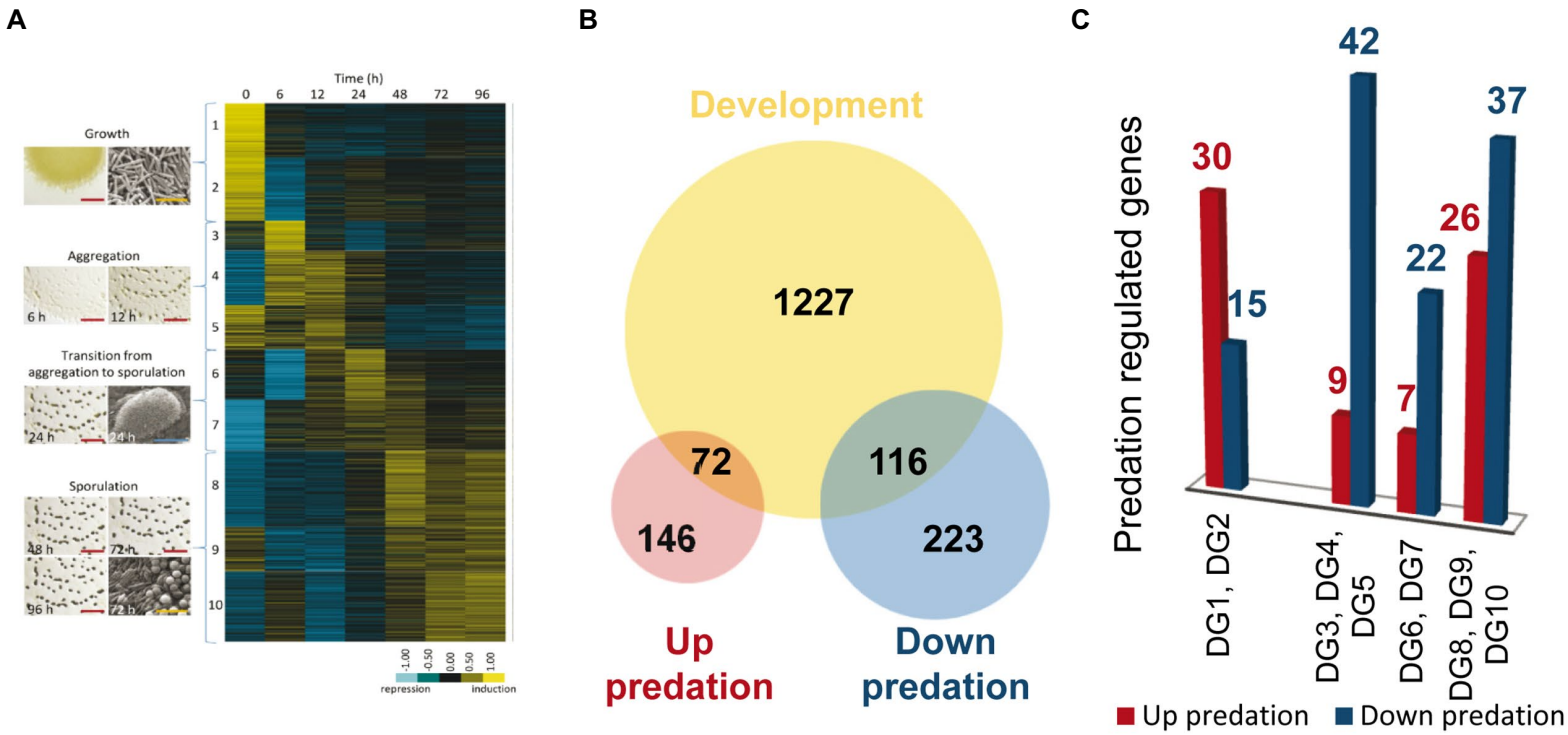
Differentially expressed genes (DEGs) during predation clustered in developmental groups (DGs) of *M. xanthus*. **(A)** DGs described by Muñoz-Dorado et al. (2019). **(B)** Venn diagram of up and down-DEGs during predation, and DEGs during development (not shown to scale). **(C)** Number of DEGs during predation included in each DG. As indicated in the text, DG1 and DG2 are downregulated genes during development, while DG3-10 are genes upregulated during development.

During development, *M. xanthus* changes the expression of 1,415 genes, which are clustered into 10 developmental groups (DGs) according to their expression profiles (Muñoz-Dorado et al., 2019). DGs 1–2 comprise genes that were downregulated upon starvation. In contrast, genes in the rest of the groups were upregulated at different stages of the developmental program. DGs 3–5 included genes required for aggregation, DGs 6–7 contained genes that are necessary during the transition from aggregation to sporulation, while genes in DGs 8–10 were associated to the sporulation phase. It should be mentioned that DG5 and DG9 contain genes that are expressed at high levels during growth, but they are later upregulated during aggregation and sporulation, respectively (Figure 6A).

To compare the transcriptomes of predation versus development we decided to use only those genes that are up and downregulated during predation included in DEGs with |Log2 Fold Change |> 1, to avoid noise that may interfere with the identification of the most relevant genes required in both processes. Out of the 557 genes with expression levels during predation over this threshold, 218 are upregulated and 339 are downregulated (Figure 6B).

A total of 72 out of 218 upregulated genes during predation are also found in the groups of development (Figures 6A,C; Supplementary Table S4A). Thirty transcripts hit in DGs1 and 2, which contain genes that are downregulated during development. Among these genes, it is noticeable to mention those that encode hydrolytic enzymes with peptidase and protease activity, which are expected to be specifically required for predation, but not for development. In contrast, most of the genes encoding proteins with glycosyl hydrolase, glycosyl transferase and peptidase activity that are upregulated during development remain at constant levels or are downregulated during predation (Supplementary Table S4B). These proteins most likely participate in the recycling of polysaccharides and proteins present in vegetative cells that are not required during development. Therefore, they are hydrolyzed to yield monomers that can be used to build specific components of the myxospores, especially of the spore coat. Of special mention is the case of the protease PopC, which is responsible for the cleavage of the p25 precursor, encoded by the gene *csgA,* to originate the C-signal p17 (Lobedanz and Søgaard-Andersen, 2003; Rolbetzki et al., 2008). As previously described C-signal production and transmission is essential to build mature fruiting bodies filled with myxospores (Kroos and Kaiser, 1987).

On the other hand, some genes upregulated during predation are also upregulated in early times of development. These are the cases of some characterized genes such *ndk* (DG1), involved in the stringent response (Muñoz-Dorado et al., 1990; Bretl and Kirby, 2016), the A-signal regulator *rpoD* (DG5; Inouye, 1990), and the B-signal related genes *bcsA* (DG2), *hsfA* and *hsfB* (DG4; Ueki and Inouye, 2002, 2003; Cusick et al., 2015). These results indicate that both A and B signal are also required for predation, and they agree with the fact that some mutants in early developmental genes are also affected in predation (Pham et al., 2005). It is interesting to recall that the A signal consists of a mixture of amino acids released by proteases that functions as a quorum-sensing mechanism to determine whether the cell density of the myxobacterial population is sufficient to successfully culminate the formation of fruiting bodies filled of myxospores. It is plausible to speculate that *M. xanthus* upregulates the expression of the same genes with hydrolytic activity during both predation and development to either optimize the scavenging of nutrients upon starvation or to kill the prey during predation. Moreover, a total of 26 genes that are upregulated during predation cluster into the late developmental groups DGS 8-10, associated with sporulation, such as *aceA* and *aceB* (Orlowski et al., 1972; Müller et al., 2010). Some of these genes are involved in lipid metabolism and secondary metabolites biosynthesis, such as homospermidine lipids or geosmin (Figures 6A,C; Supplementary Table S4A). The role of these genes in both processes remains to be elucidated, although in the case of the biosynthesis of secondary metabolites, it is plausible to think that they may be used to kill the prey during predation or to protect the myxospores inside the fruiting bodies from other competitors during development, helping to generate those nutrients that will trigger germination.

Many upregulated genes during predation at 6 h are devoted to ribosomal biosynthesis and translation (Section “Ribosomal biogenesis and translation”; Supplementary Figure S4B). It is worth to mention that most of those genes are downregulated at 6 h of development, but subsequently upregulated to a level like (or even higher) that observed during growth (Muñoz-Dorado et al., 2019). An interesting result is the expression profiles of the two paralogs for ribosomal protein S4, MXAN_6908 and MXAN_3325. During development, MXAN_3325 drastically increases its expression, while MXAN_6908 decreases (Muñoz-Dorado et al., 2019). However, during predation both paralogous are upregulated. These observations point to the direction that regulation of the translational machinery (in general) and the protein S4 (in particular) play important roles in *M. xanthus* lifecycle that remain unexplored.

On the other hand, 116 genes that are downregulated during co-culture are also found in the DGs (Figures 6B,C). Most of these genes appear in DGs 3–10, indicating that they are upregulated during aggregation or sporulation. Among these genes, the major global regulators *fruA* and *mrpC* are found (Supplementary Tables S2B, S4B). Moreover, other genes such as *espA* and *espC*, which regulate the developmental timing, *treY*, related to sporulation, the gene for the alternative sigma factor SigC, *relA*, essential for the stringent response, the E-signal related genes *esgA* and *esgB*, and others from the C-signal pathway, such as *popC*, *popD*, *sdeK, fmgB*, *nla6, cbgA*, and *hsfB*, involved in sporulation are also upregulated during development but downregulated during predation (see references in Supplementary Tables S2B, S4A). Another noteworthy result is the downregulation of the Che6 and Che7 chemosensory systems (Supplementary Figure S5B; Supplementary Tables S2B, S4B). Che6 system seems to be implicated in PilA assembly, while Che7 system is involved in motility, aggregation and sporulation, and it seems to affect membrane composition (Zusman et al., 2007; Kirby et al., 2008).

As conclusions, the *M. xanthus* predatosomes so far available, although obtained using different laboratory conditions, indicate that the type of hydrolytic enzymes and secondary metabolites produced by *M. xanthus* differ depending on the prey/culture conditions. In contrast, lipid metabolism, iron uptake and motility seem to be relevant pathways important for *M. xanthus* predation in general. Moreover, while during co-culture the predator upregulates the expression of genes that help to kill, lyse, and consume the prey, downregulates many regulatory genes, most of which have been demonstrated play key roles in development, indicating that the transcriptional regulation exerted by *M. xanthus* on certain genes helps to choose between entering the developmental cycle or continuing in a vegetative stage. The results shown here have shed some light on the transcriptional regulation during the entire *M. xanthus* lifecycle and confirm that this multicellular bacterium uses exceptional and complex mechanisms for the integration of development and predation.

## Data availability statement

The datasets presented in this study can be found in online repositories. The names of the repository/repositories and accession number(s) can be found at: https://www.ncbi.nlm.nih.gov/, PRJNA860082.

## Author contributions

JP: substantial contributions to conception, design, analysis, interpretation of data, draft the article and revising it critically for important intellectual content; funding acquisition. FC-M: acquisition, analysis and interpretation of data, and editing and revising the article critically for important intellectual content. JM-D: substantial contributions to conception, design and interpretation of the data, and revising the article critically for important intellectual content; funding acquisition. AM-M: acquisition and analysis of data, editing and revising the article critically for important intellectual content; funding acquisition. All authors contributed to the article and approved the submitted version.

## Funding

This research was funded by the Spanish Government (grant no. PID2020-112634GB-I00) and FEDER funds (grant no. A-BIO-126-UGR20).

## Conflict of interest

The authors declare that the research was conducted in the absence of any commercial or financial relationships that could be construed as a potential conflict of interest.

## Publisher’s note

All claims expressed in this article are solely those of the authors and do not necessarily represent those of their affiliated organizations, or those of the publisher, the editors and the reviewers. Any product that may be evaluated in this article, or claim that may be made by its manufacturer, is not guaranteed or endorsed by the publisher.
