# Supplementary material for: Development versus predation: Transcriptomic changes during the lifecycle of Myxococcus xanthus
Source: Front Microbiol. 2022 Sep 26;13:1004476. doi: 10.3389/fmicb.2022.1004476 (PMC9548883; doi:10.3389/fmicb.2022.1004476)
Supplement: Supplementary file 1 [file Data_Sheet_1.PDF]

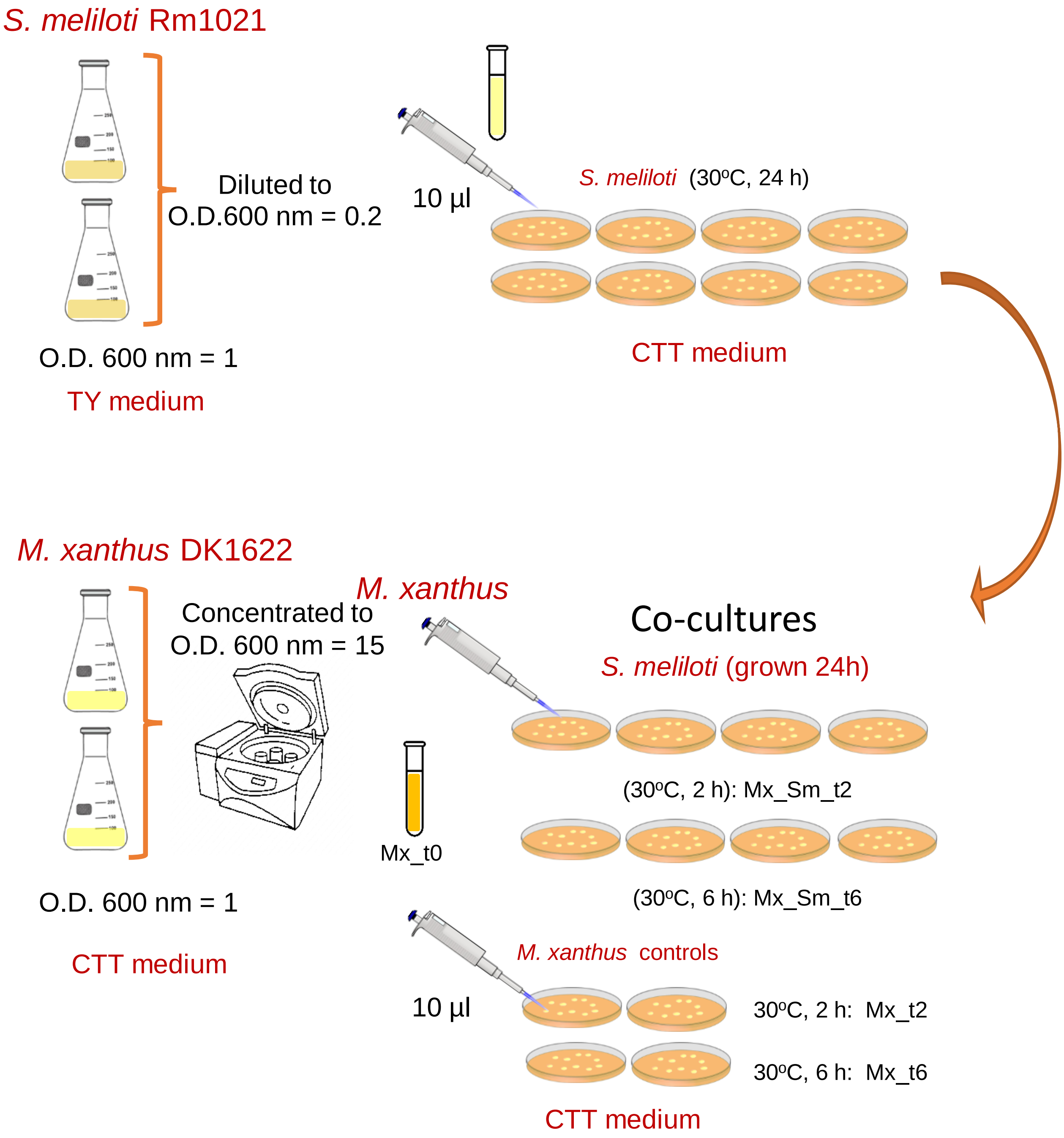

Figure S1. **Methodology used to obtain synchronous samples of *M. xanthus* preying on *S. meliloti*.** *S. meliloti* Rm1021 was grown in solid medium and incubated for 24 hours. After this time, drops of *M. xanthus* DK1622 were deposited over the *S. meliloti* cultures. Controls were collected at 0, 2 and 6 hours, whereas cell mixtures were harvested after 2 and 6 hours of interaction. RNA and cDNA were obtained for RNA-seq massive sequencing by using Illumina NovaSeq6000.
