# Supplementary material for: Development versus predation: Transcriptomic changes during the lifecycle of Myxococcus xanthus
Source: Front Microbiol. 2022 Sep 26;13:1004476. doi: 10.3389/fmicb.2022.1004476 (PMC9548883; doi:10.3389/fmicb.2022.1004476)
Supplement: Supplementary file 2 [file Data_Sheet_2.PDF]

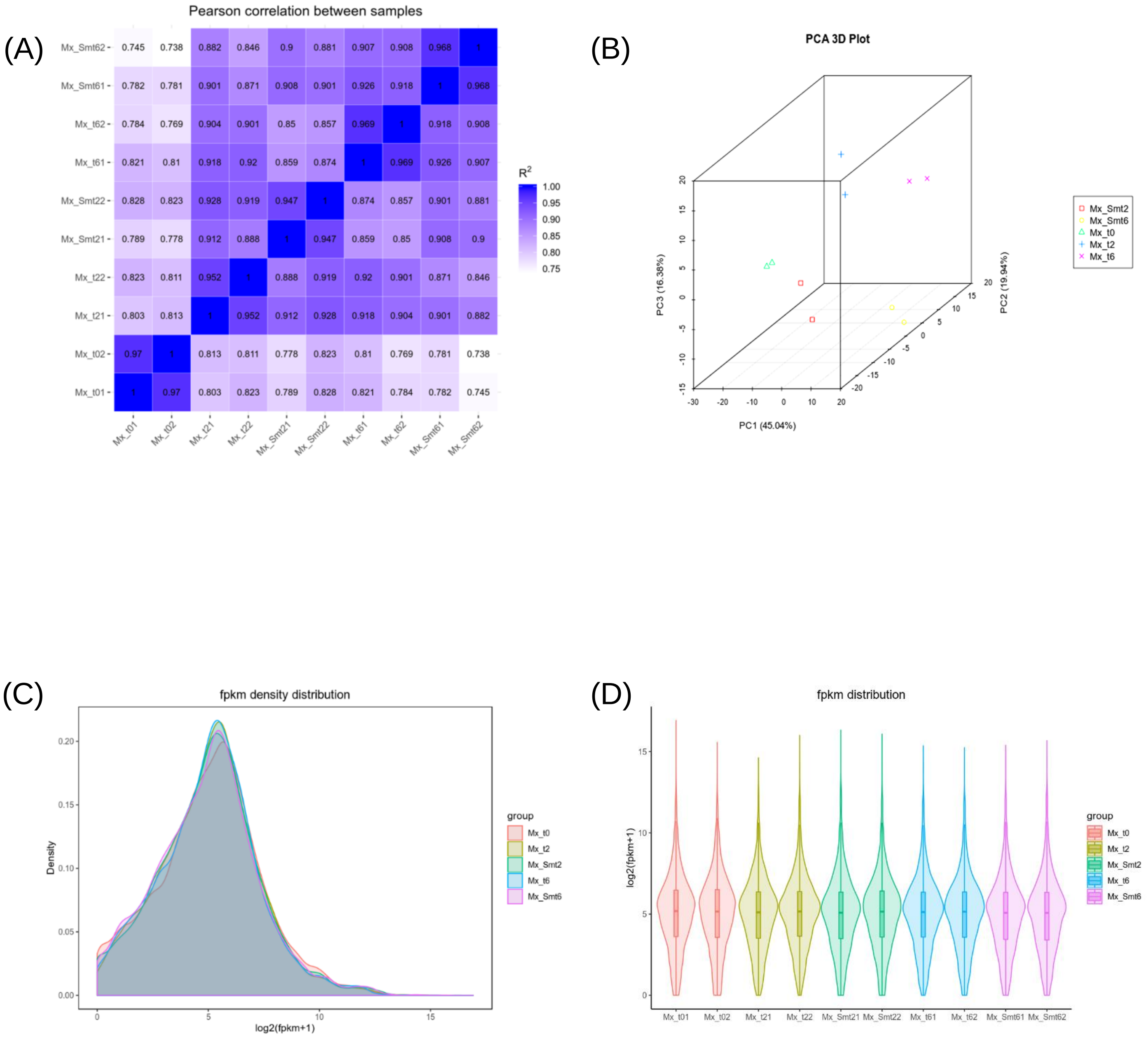

Figure S2. **Correlation between samples and comparisons of the FPKM values between samples.** (**A**) Pearson correlation. t0, t2 and t6 means 0, 2 or 6 hours of *M. xanthus* pure culture (Mx) or interaction with *S. meliloti* (Mx_Sm), respectively. Two replicates were performed for each condition. (**B**) Principal component analysis (PCA) of RNA-seq. Transcriptomic (global gene expression) data from each of the time points were analyzed by principal component analysis using R statistical language. Colored dots represent individual biological replicates harvested at the different times and conditions. **(C)** FPKM density distribution. The x-axis shows the log10(FPKM+1) and the y-axis shows gene density. (**D**) FPKM violin plot. The x-axis shows the sample names and the y-axis shows the log10(FPKM+1). Each violin has five statistical magnitudes (maximum value, upper quartile, median, lower quartile, and minimum value). The violin width shows the gene density.
