# Supplementary material for: Development versus predation: Transcriptomic changes during the lifecycle of Myxococcus xanthus
Source: Front Microbiol. 2022 Sep 26;13:1004476. doi: 10.3389/fmicb.2022.1004476 (PMC9548883; doi:10.3389/fmicb.2022.1004476)
Supplement: Supplementary file 3 [file Data_Sheet_3.PDF]

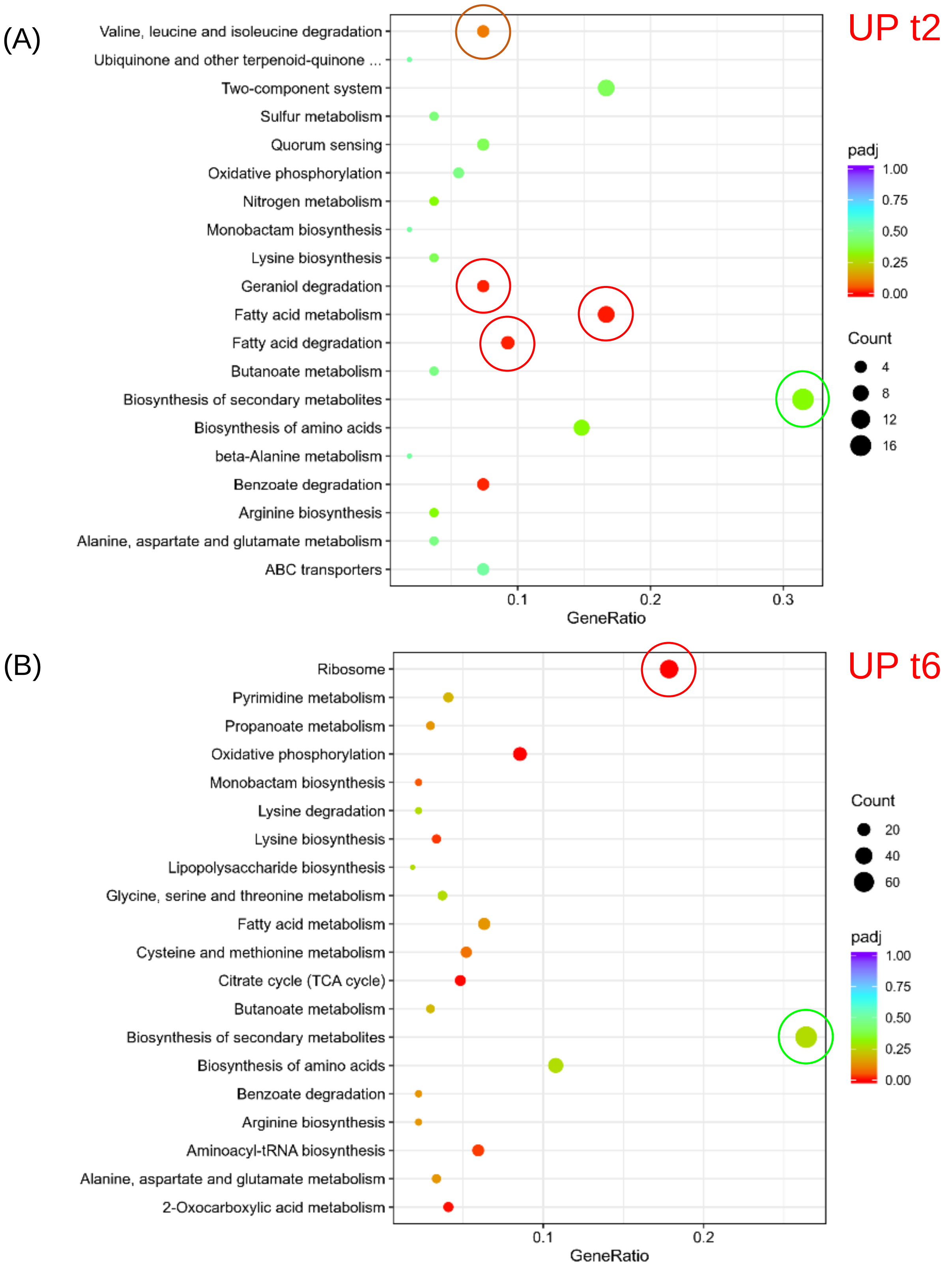

Figure S3A. **Enrichment of genes upregulated during predation in significant KEGG pathways.** *M. xanthus* in predatory conditions versus *M. xanthus* in pure culture at time 2 and 6 hours (t2 and t6) and enrichment during predation (from t2 to t6). **(A)** Predation time 2 hours (t2), Mx_Smt2vsMxt2 and **(B)** Predation time 6 hours (t6), Mx_Smt6vsMxt6. The y-axis shows the KEGG pathway modules and the x-axis shows the ratio of differentially expressed genes to all genes concerned to this KEGG pathway module. In red, green, and brown are the modules described in the main text. See Supplementary Table S1E and S1FG for gene details.

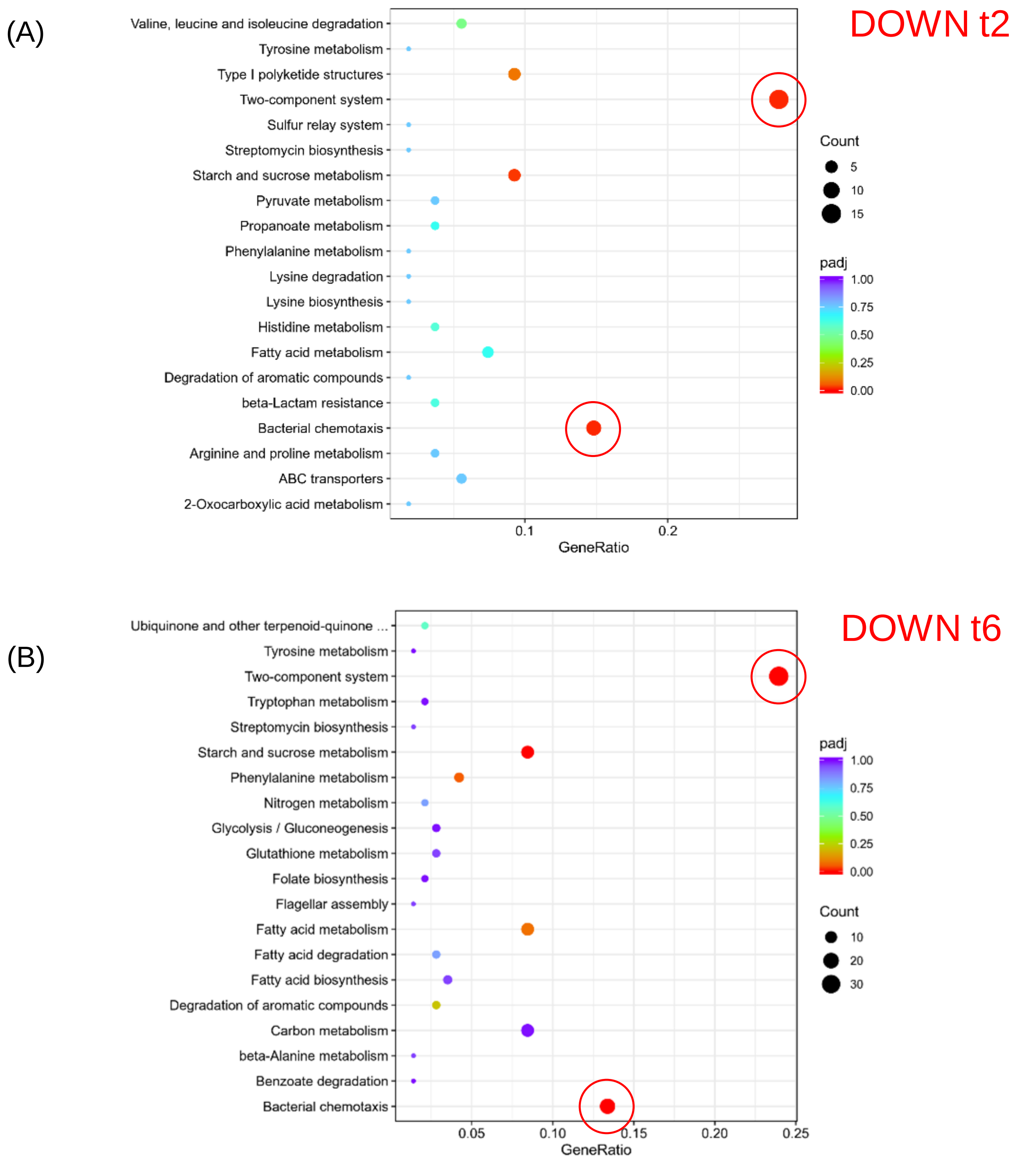

Figure S3B. **Enrichment of genes downregulated during predation in significant KEGG pathways.** *M. xanthus* in predatory conditions versus *M. xanthus* in pure culture at time 2 and 6 hours (t2 and t6) and enrichment during predation (from t2 to t6). **(A)** Predation time 2 hours (t2), Mx_Smt2vsMxt2 and **(B)** Predation time 6 hours (t6), Mx_Smt6vsMxt6. The y-axis shows the KEGG pathway modules and the x-axis shows the ratio of differentially expressed genes to all genes concerned to this KEGG pathway module. In red are the modules with padj≤0.05. In red circles are the modules described in the main text. See Supplementary Table S1I and S1J for gene details.
