# Supplementary material for: Development versus predation: Transcriptomic changes during the lifecycle of Myxococcus xanthus
Source: Front Microbiol. 2022 Sep 26;13:1004476. doi: 10.3389/fmicb.2022.1004476 (PMC9548883; doi:10.3389/fmicb.2022.1004476)
Supplement: Supplementary file 4 [file Data_Sheet_4.PDF]

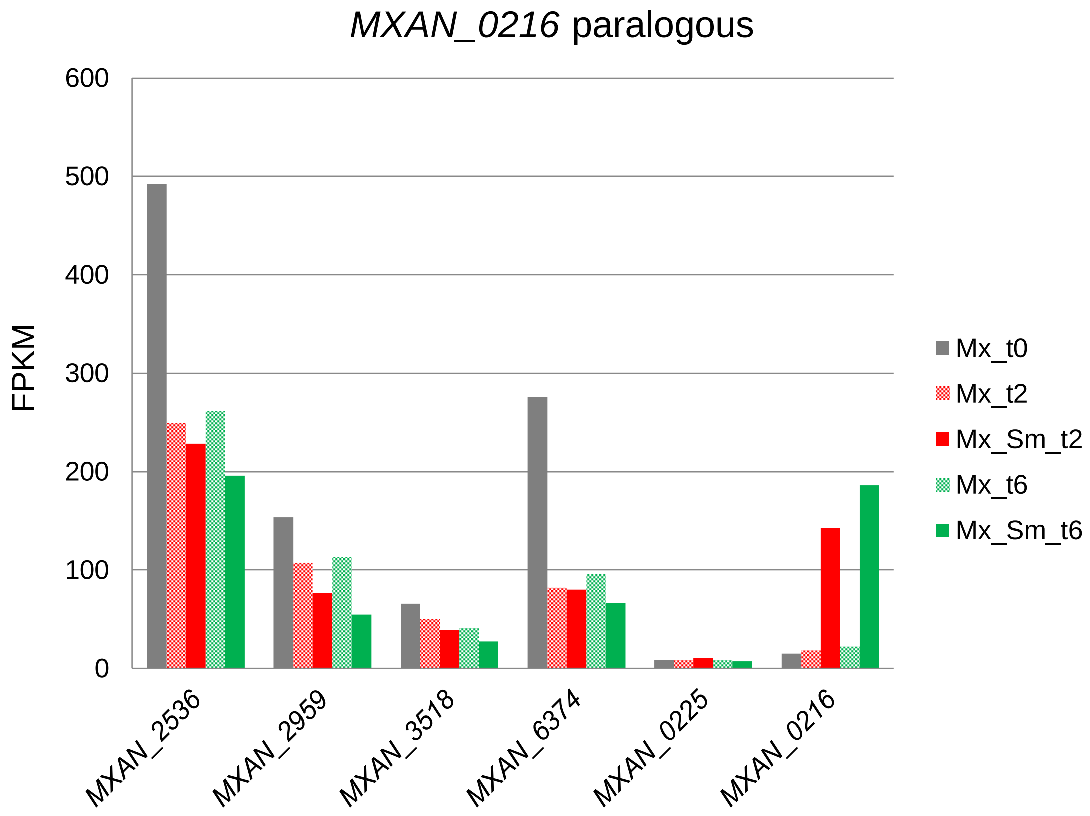

Figure S4A. **Putative long-chain fatty acid-CoA ligase paralogous in *M. xanthus* genome.** Only *MXAN_0216* is upregulated during predation.

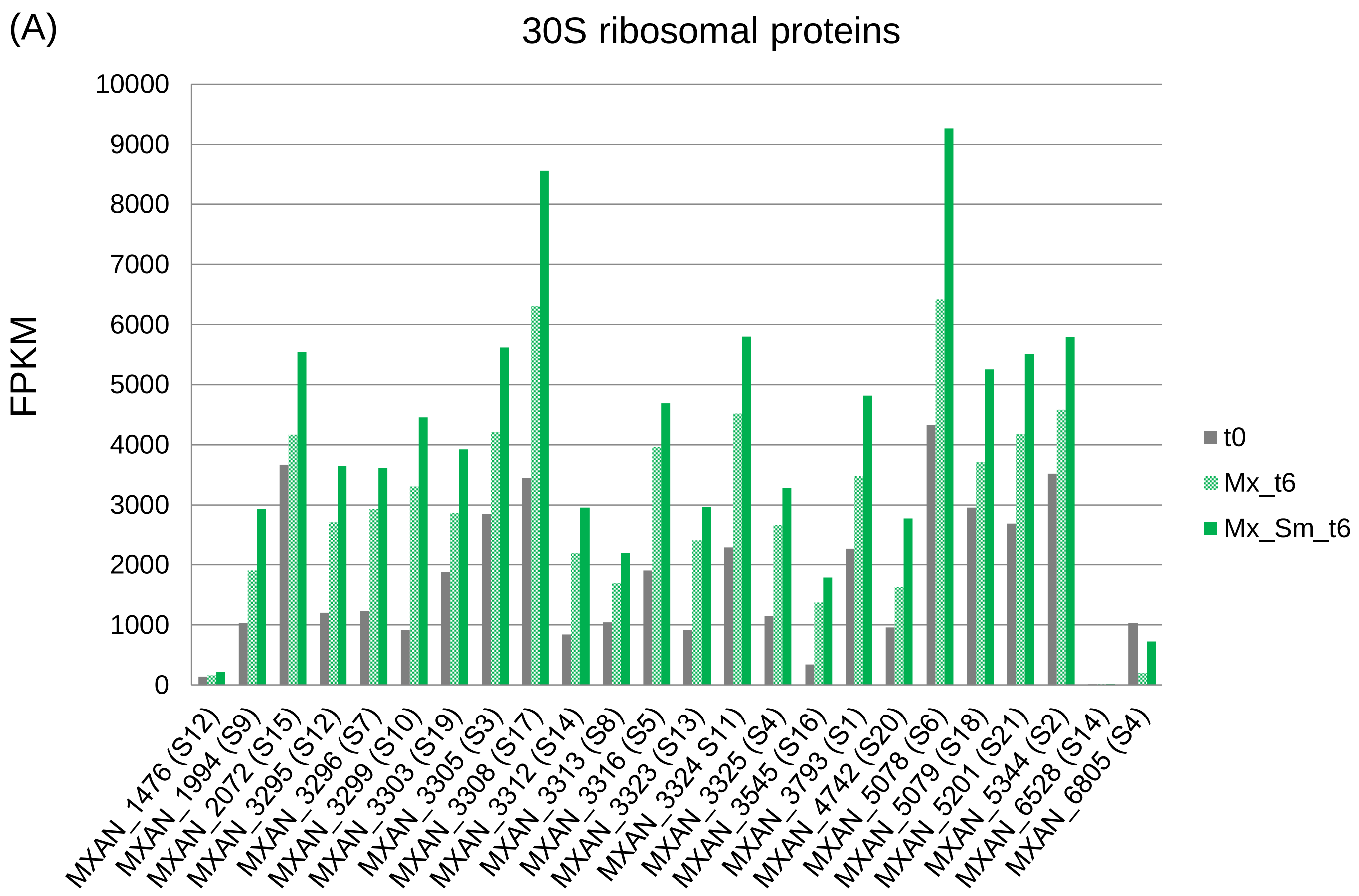

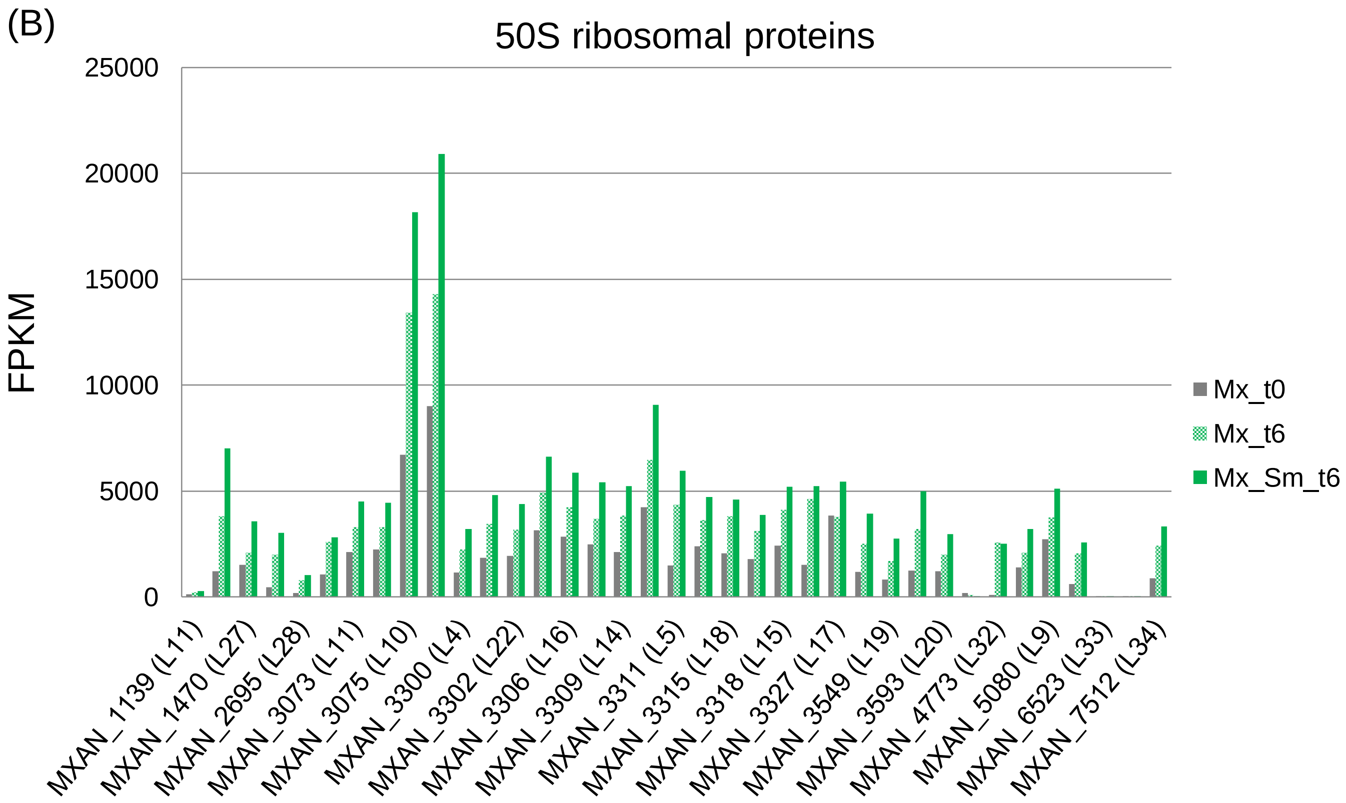

Figure S4B. **Expression of genes coding for (A) 30S and (B) 50S ribosomal proteins** at time 0 and 6 in *M. xanthus* pure culture (Mx_t0 and Mx_t6, respectively), and at time 6 in *M. xanthus* co-colture with *S. meliloti* (Mx_Sm_t6).
