# Supplementary material for: Development versus predation: Transcriptomic changes during the lifecycle of Myxococcus xanthus
Source: Front Microbiol. 2022 Sep 26;13:1004476. doi: 10.3389/fmicb.2022.1004476 (PMC9548883; doi:10.3389/fmicb.2022.1004476)
Supplement: Supplementary file 5 [file Data_Sheet_5.PDF]

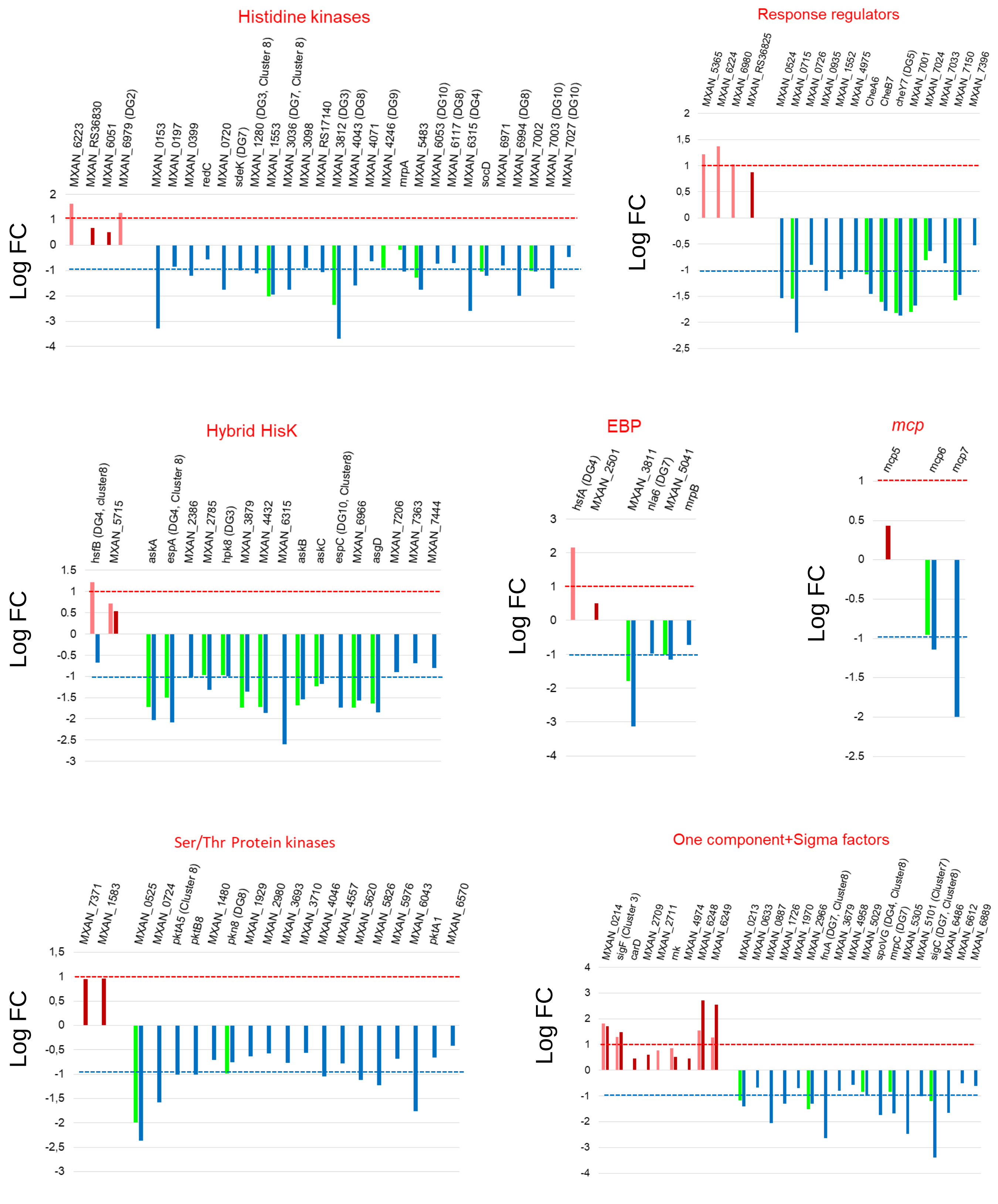

Figure S5A**. Diferentially expressed families of regulatory proteins during predation.** In pink and green, LogFC at t2; in red and blue, LogFC at t6. The dashed lines mark the threshold of |Log2 Fold Change|>1.

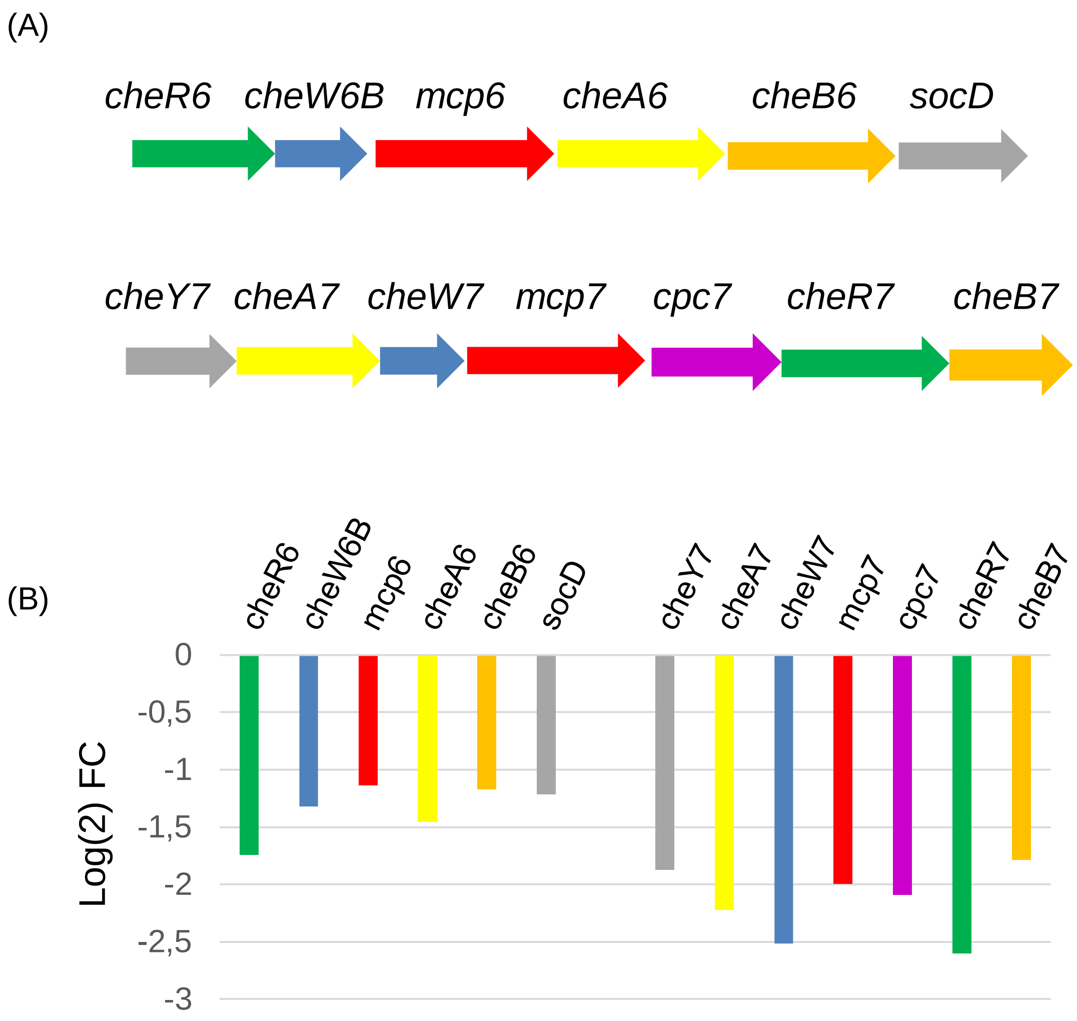

Figure S5B. **Downregulation at early times of predation of the Che6 and Che7 chemosensory systems**. **(A)** Genetic organization of Che6 and Che7 chemosensory systems in *M. xanthus* DK1622 genome. **(B)** Downregulation of all the genes of both operons.
